# Coding in Primary Grades Boosts Children’s Executive Functions

**DOI:** 10.3389/fpsyg.2019.02713

**Published:** 2019-12-11

**Authors:** Barbara Arfé, Tullio Vardanega, Chiara Montuori, Marta Lavanga

**Affiliations:** ^1^Department of Developmental Psychology and Socialization, University of Padova, Padua, Italy; ^2^Department of Mathematics, University of Padova, Padua, Italy

**Keywords:** coding, computational thinking, programming, executive function, primary school children

## Abstract

Several programs have been developed worldwide to improve children’s executive functions (EFs). Yet, the role played in EF development by learning activities embedded in the school curriculum has received scarce attention. With two studies, we recently tested the effects of *computational thinking* (CT) *and coding*—a new element of the primary school curriculum—on the development of children’s EFs. CT stimulates the ability to define a clear and orderly sequence of simple and well-specified steps to solve a complex problem. We conjecture that CT skills are associated to such EF processes as response inhibition and planning. In a first between-group cluster-randomized controlled trial, we tested the effects of 1-month coding activities on 76 first graders’ planning and response inhibition against those of 1-month standard STEM activities of a control group. In a second study, we tested the effects of 1-month coding activities of 17 second graders in two ways: within group (longitudinally), against 7 months of standard activities experienced by the same children (experimental group); and between groups, in comparison to the effects of standard STEM activities in a control group of 19 second graders. The results of the two studies show significant benefits of learning to code: children exposed to coding improved significantly more in planning and inhibition tasks than control children did. The longitudinal data showed that improvements in planning and inhibition skills after 1 month of coding activities (eight lessons) were equivalent to or greater than the improvement attained after 7 months of standard activities. These findings support the hypothesis that learning CT *via* coding can significantly boost children’s spontaneous development of EFs.

## Introduction

Between the ages of 5 and 7, in the transition period from preschool to primary school, children undergo rapid changes in their cognitive functioning (Roebers et al., 2011; Traverso et al., 2015; Vandenbroucke et al., 2017). The product of these changes, i.e., their resulting executive functioning (EF), has long-lasting effects on their future academic achievements and self-regulation skills (Altemeier et al., 2006; Friedman et al., 2014; Blair, 2016; Schmitt et al., 2017; Escobar et al., 2018; Stad et al., 2018). Interventions to enhance executive functions (EFs) in this time window thus are extremely important. The scientific literature suggests that the training of EFs has wider benefits if implemented early (Diamond et al., 2007; Espinet et al., 2013; Traverso et al., 2015; Blair, 2017) and if embedded in children’s everyday activities (Traverso et al., 2015; Diamond and Ling, 2016; Blair, 2017).

Several studies have been conducted in the last few years to test the contribution of early intervention on the development of EFs (Liu et al., 2015; Traverso et al., 2015; Howard et al., 2018; Zhang et al., 2019). Other studies have explored the efficacy of *ad hoc* EF training programs (Kronenberger et al., 2011; Espinet et al., 2013; Grunewaldt et al., 2013; Hardy et al., 2016; Aarnoudse-Moens et al., 2018; Boivin et al., 2019; Zhang et al., 2019). For a review of intervention programs, see Diamond and Ling (2019). To date, however, the role played by everyday curriculum-based, learning activities on children’s EFs has received scarce attention.

This paper addresses this gap by examining the effects of a new curriculum-based activity (coding) on first and second graders’ EFs. Coding (i.e., programming) is the instrumental skill of computational thinking (CT), broadly referred to as the set of problem-solving processes that underlie the solution of computational problems (i.e., those whose solution can be performed by a computing agent) (Wing, 2006; Roman-Gonzalez et al., 2017). Although related to an approach to problem-solving that is proper of computer science (Wing, 2006; Nardelli and Ventre, 2015; Florez et al., 2017), CT can be conceived as a general way of thinking of problems, and thus it can be generalized to various types of problems that do not directly involve programming tasks or computers (Wing, 2006). Coding is the prime means used to teach CT in primary schools (Lye and Koh, 2014; Nardelli and Ventre, 2015; Saez-Lopez et al., 2016; Roman-Gonzalez et al., 2017; Tuomi et al., 2018).

### Testing the Effects of Coding on EF

Some studies have focused on the general effects of schooling on EFs (Brod et al., 2017; Zhang et al., 2019). Yet, very few studies have examined the association between specific curriculum-based activities at school (e.g., literacy activities) and EFs (Diamond et al., 2007; Burrage et al., 2008; Baker et al., 2015). Except for a few notable exceptions (e.g., Diamond et al., 2007; Blair and Raver, 2014), such studies did neither apply a randomized controlled trial design (Baker et al., 2015) nor compare children of the same level of instruction (Burrage et al., 2008). For example, Burrage et al. (2008) compared pre-kindergarten to kindergarten children of the same age, the former waiting to enter the kindergarten, the latter attending it. Thus, their study lacked a comparison condition in which the specific literacy activity (e.g., letter and word reading) had not been introduced yet in the curriculum at that grade level (kindergarten).

The problem in determining the benefits for EFs drawn from specific learning activities in school is that no control groups (i.e., children who lack the relevant experience) typically exist: All children learn to read and write, though with alternate success. However, the recent introduction of CT, and with it, of coding in the primary school curriculum in Europe and the United States, provides the opportunity to test the effects of a new curriculum-based learning activity on children’s EFs.

Computational thinking involves a set of higher order cognitive abilities, such as (1) to analyze problems and decompose them in smaller parts; (2) to plan a sequence of steps or instructions for the solution of each sub-problem, intended for the execution by either a computer or a human agent; (3) to recognize errors in the solution, and fix them (i.e., debugging); (4) to generalize or apply the problem-solving strategies learnt to different contexts and other kinds of problem-solving tasks (Wing, 2006; Shute et al., 2017). Owing to its being a problem-solving process, CT makes significant demands on the individual’s EFs, requiring a significant extent of working memory capacities (Shute et al., 2017), response inhibition (Di Lieto et al., 2017), and planning (Chao, 2016). Conceivably, therefore, guided experience of CT problems, through coding activities in school, might boost children’s EFs significantly.

In several countries, including Italy, children enter school with no prior or very limited knowledge of coding. While spreading worldwide, coding instruction is not yet adopted in all schooling institutions and classroom laboratories. These circumstances allow researchers to explore the effects of this specific learning activity on children’s cognitive skills and EFs.

### The Teaching of Coding in Primary School

The state-of-the-art literature in this field suggests that several approaches and tools can be used to teach coding in primary schools (Florez et al., 2017), with block-based visual programming, like Scratch^1^ (Resnick et al., 2009; Saez-Lopez et al., 2016) or Code.org^2^ (Kalelioglu, 2015), seen as the most effective for preschoolers and children beginning primary school (Saez-Lopez et al., 2016). The two studies presented in this paper used resources from Code.org to train the coding skills (and EFs through them) of Italian children in first and second grades.

Code.org is an open-source programming platform launched by the Code.org non-profit to expand access to computer science in schools among young children (Kalelioglu, 2015; Nardelli and Ventre, 2015), and to increase participation to it by under-represented gender and social minorities. Coding exercises on Code.org employ intuitive drag-and-drop applications and block-based visual language, particularly appropriate for young learners (Kalelioglu, 2015; Saez-Lopez et al., 2016). The platform provides engaging scenarios for children of different age and gender, and personalized feedback, which allow tailoring the pedagogical experience to the individual child. The teaching of coding may involve plugged (computer based) and unplugged (e.g., paper and pencil) learning activities, whose common goal is to introduce children to problem-solving through programming. Children are introduced to a programming language (prevalently block-based and visual) and to the use of the logical operators involved in developing a program, such as sequencing (defining a sequence of steps to achieve a goal), or debugging (locating errors in the program and correcting them). A program is operatively defined to children as any sequence of instructions that guide an artificial agent (a computer) or a fellow human to achieve a stated goal. Thanks to the accessibility of resources like Code.org or Scratch, instructional coding activities are slowly spreading across schools. Yet, the schools in which coding has been regularly embedded in the STEM curriculum are still few, and most teachers lack familiarity with coding resources as well as with the instructional basics to introduce their classrooms to coding.

To the best of our knowledge, the only study that has investigated the cognitive effects of Code.org activities at primary school (Kalelioglu, 2015) exposed fourth graders to a 5-h course (1 h per week) through the Code.org platform. Kalelioglu (2015) assessed the effects of that learning activity on children’s reflective thinking toward problem-solving. Such trial found no evidence of significant positive effects of coding on it. Yet, the ability assessed by Kalelioglu (2015) (reflective thinking, which is part of critical thinking) might arguably be too complex for fourth graders, and with insufficient sensitivity to the nuances in cognitive changes induced by the coding activities at that age.

In the two studies presented in this paper, we tested the effects of coding (problem-solving) activities selected from Code.org on 5- to 6-year-old children’s planning and response inhibition skills. Those two EFs are especially interesting as their development undergoes substantial changes from preschool to the first years of primary school (Davidson et al., 2006; Diamond, 2006; Magi et al., 2016; Zelazo et al., 2016; Di Lieto et al., 2017). We also show how 1 month of *ad hoc* designed coding activities in second grade can produce a greater improvement in these EFs than that observed in the same children after 7 months of regular curriculum and learning activities.

The teaching of coding involves the ability to analyze problems and to conceive algorithmic procedures (i.e., plans) for their solution (Florez et al., 2017). Given the role played by planning in (computational) problem-solving (Chao, 2016; Chen et al., 2017), we believe that the cognitive ability to plan can be scaffolded and enhanced by appropriate CT activities in the class. For instance, putting individual program instructions into an ordered sequence, a key methodical skill of CT, does involve working memory and planning, that is, the ability to organize a sequence of actions in a manner apt to achieve a given goal (Bers et al., 2014). Moreover, as analyzing the problem space to devise a multi-step plan also requires cognitive control over immediate and impulsive responses (Luciana et al., 2009; Wang and Chiew, 2010; Magi et al., 2016), we conjecture that learning to code—to solve computational problems—may also foster the development of children’s response inhibition skills. Some preliminary evidence (Di Lieto et al., 2017) suggests the association between coding and the development of inhibition skills in young children (aged 5–6 years). Di Lieto et al. (2017) demonstrated the positive effects of programming in a tangible environment (one in which children interact with physical objects, robots, in a physical space, e.g., a room), on the working memory and inhibition skills of a group of 12 5–6-year-old preschoolers. Being tangible, that is, concrete, the learning environment of educational robotics is deemed particularly appropriate to stimulate the cognitive skills of preschoolers and young primary school children (Wyeth and Wyeth, 2001; Bers et al., 2014; Shim et al., 2017). Our studies extend the findings of Di Lieto et al. (2017) by examining whether also *virtual* learning environments, such as those provided by the Code.org platform, can be effective in improving 5–6-year-old children’s EFs, i.e., planning and inhibition skills.

As noted above, transition to school is a particularly sensitive period for the development of EFs (Roebers et al., 2011; Macdonald et al., 2014; Magi et al., 2016; Poutanen et al., 2016). Recently, Macdonald et al. (2014) observed that response inhibition skills develop rapidly in the early school years, from the age of 5 to 7. Also, planning skills seem to develop significantly in the first years of schooling (Magi et al., 2016; Poutanen et al., 2016) and their development relate significantly to that of reading and math skills (Crook and Evans, 2014; Magi et al., 2016). Thus, interventions designed to boost the development of response inhibition and planning can be particularly effective in this time window. Delivered at this age, they also may have positive impact on other school achievements.

## Study 1

Study 1 addressed the following two research questions:

(1) Can a short training with coding (4 weeks) through Code.org enhance the planning and response inhibition skills of first graders? Based on prior research (Di Lieto et al., 2017), we anticipated that learning to code would affect positively both planning and response inhibition, increasing planning time and accuracy on standardized planning tasks, and contributing to decrease inhibition errors and inhibition time on standardized inhibition tasks.
(2) Are the positive effects of such training retained at 1 month from the end of the intervention? We predicted that positive training effects would be maintained.

**FIGURE 1:**
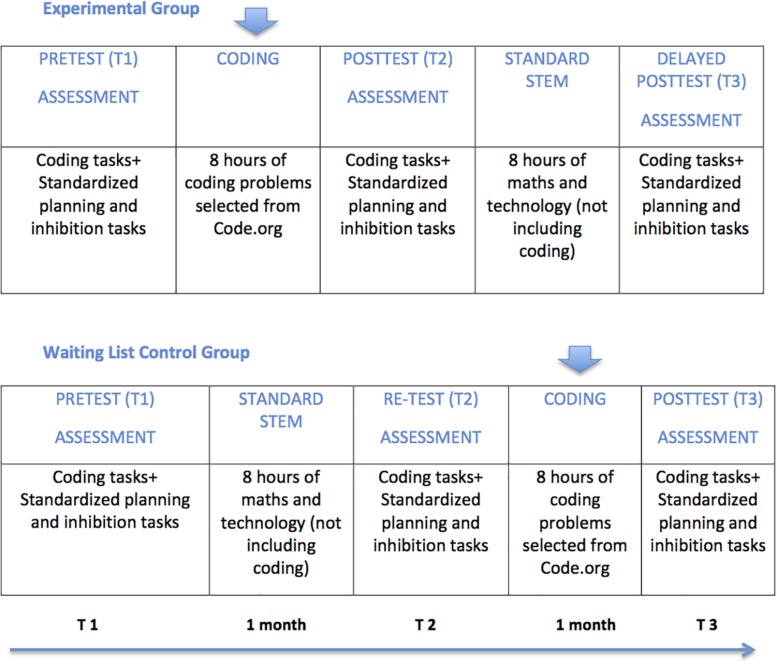
Experimental design study 1.

We performed a cluster-randomized controlled trial (Campbell et al., 2012) to test the effects of exposure to Code.org activities. Four classrooms of first graders (80 children) were randomly assigned to an experimental condition (coding) or control condition (waiting list), based on a matched design procedure. Classrooms were matched in pairs on gender distribution, age, socio-economic status (SES), and for teachers (i.e., each classroom pair had the same team of teachers), and then randomly assigned to either coding training or the waiting-list condition. The coding abilities, planning skills, and response inhibition skills were tested before (pre-test, T1) and after (post-test, T2) the coding intervention, as well as at 1-month distance from the training (delayed post-test, T3). The waiting-list group received the coding intervention after the post-test (T2); hence, the assessment at T3 was the post-test for this group (see Figure 1).

### Participants

Eighty 5–6-year-old children at the beginning of first grade participated in the study. The experimental group included 44 first graders (20 girls, 45%, 24 boys, 54%, mean age 6.07). The waiting-list group consisted of 36 first graders (21 girls, 58%, 15 boys, 42%, mean age 5.9). None of those children needed or received treatment for learning disabilities or developmental disorders. All were native Italian speakers. Parental written informed consent was collected before the study for all participants. The study was approved by the Ethical Committee of the Department of Developmental Psychology (University of Padova, Italy). Demographic data for the experimental and waiting control group are reported in Table 1.

**TABLE 1:** Study 1: Demographic characteristics of the experimental and waiting group.

	**Experimental**	**Waiting**	***p-*value**
**Gender**			
Girls (*n*, %)	19, 45%	20, 59%	0.25
Boys (*n*, %)	23, 55%	14, 41%	
Age (*M*, *SD*)	6.05 (0.58)	5.97 (0.46)	0.53
SES (*M*, *SD*)	6.14 (1.42)	5.71 (1.73)	0.23

#### Socio-Economic Status

As children’s ability to benefit from coding can be mediated by low SES (Israel et al., 2015) and SES is associated with poorer EF skills and school achievement in STEM (Blair and Raver, 2016; Blums et al., 2017), the SES of the two groups was assessed to make sure they not differ on this variable. Socio-economic data were collected through a socio-demographic questionnaire that parents returned with the written informed consent to the study. Children’s SES was estimated based on parents’ education (from 0, less than elementary school, to 4, college) and occupation (from 1, unemployed, to 4, professional roles). A composite score was calculated as the sum of the highest education score and the highest occupation score obtained by either parent (Arfé et al., 2018), with a maximum score of 8.

### Procedure and Materials

We used a selection of Code.org coding problems for training (Arfé et al., under review). With Code.org, children move blocks of basic instructions (code) to generate sequences of commands that instruct a sprite (e.g., an angry bird) to perform actions, in the intent to achieve a given goal. The platform provides visual and written informative feedback upon execution. Task difficulty increases progressively as children improve in coding, so that children face coding trials of rising difficulty: e.g., sequences, loops, and conditional instructions. The overall lesson plan involved eight coding sessions (two lessons a week for 4 weeks) and was designed to cause children to switch computing functions or scenarios frequently, to maintain a problem-solving approach to the coding tasks. Course 1 of the Code.org platform “Programma il futuro”^3^ was used, as our participants were beginning readers.

Children worked alone at their computer in a laboratory. A post-graduate student, trained by the first and second author of this study, conducted the coding lessons. Each coding lesson lasted about 60 min, and involved the execution of five to eight coding problems (see Table 2 for the full lesson plan).

**TABLE 2:** Lesson plan.

**Coding**			
**sessions**	**Course A**	**Trial number**	**Content**
Session 1	Lesson 3	1, 6	Jigsaw: Drag and Drop
	Lesson 4	2, 5, 6, 7	Maze: Sequence
Session 2	Lesson 4	8, 10	Maze: Sequence
	Lesson 5	3, 4, 5, 6, 7	Maze: Debugging
Session 3	Lesson 5	8, 9,10	Maze: Debugging
	Lesson 8	4, 5, 6, 7, 8	Artist: Sequence
Session 4	Lesson 8	9, 10, 11	Artist: Sequence
	Lesson 10	4, 5, 6, 7, 8	Artist: Shapes
Session 5	Lesson 13	1, 2, 3, 4, 5, 6, 7	Maze: Loops
Session 6	Lesson 13	8, 9, 10, 11, 12	Maze: Loops
Session 7	Lesson 14	3, 5, 6, 7, 8, 9	Bee: Loops
Session 8	Lesson 18	2, 4, 5, 6, 7	Artist: Loops
Closing session	Classroom discussion	What have we learned?	Metacognitive reflection on the goals of computational thinking and the meaning of programming

#### Pre-test, Post-test, and Delayed Post-test Assessment

##### Coding skills

At the pre-test and post-test, and at the delayed post-test, children performed four coding problems from Code.org (Course 1, Italian platform) individually: trial 9 (lesson 4), trial 2 (lesson 5), trial 3 (lesson 8), and trial 4 (lesson 14). Both the experimental and the waiting group first familiarized with the Code.org platform and the drag-and-drop mechanics, performing the first trial of lessons 4, 5, 8, and 14 from Course 1, assisted by the experimenter. The pre-test started after this familiarization phase.

For each test trial, we recorded both accuracy and planning time:

(1) *Accuracy*: a score of 2 was given if the child successfully solved the item at first attempt, 1 on solving it at the second attempt, 0 otherwise;
(2) *Time spent planning*: the seconds elapsed from the moment the child received the task instructions to the moment s/he moved the first block was recorded.

##### Planning and response inhibition skills

We used standardized tests to assess children’s response inhibition and planning at T1, T2, and T3: two tasks were used to assess inhibition and planning skills to verify whether potential benefits on EFs generalized across different tasks.

##### Planning skills

The *Elithorn maze test* (Spinnler and Tognoni, 1987) and the *Tower of London* (ToL; Luciana et al., 2009) were used to assess non-verbal planning skills.

The Elithorn maze test assesses non-verbal planning by requesting the child to trace a line on a maze to connect a number of black dots, arranged randomly on grids. Three rules are given: trace lines from the bottom up; do not cross over the grid; and do not backtrack. The overall test consists of eight mazes, each of which to be performed in no more than 2 min. Although originally standardized for Italian adolescents aged 12–18 years (BVN, Batteria per la Valutazione Neuropsicologica) (Gugliotta et al., 2009), recently the task has been used also with younger children, from the age of 6, demonstrating good sensitivity to their planning skills (Arfé et al., 2018). The children’s individual performance was scored for:

(1) *Accuracy*: i.e., the total number of mazes successfully completed within 2 min. The scoring system was 2 for each trial successfully solved within 1 min; 1 if the task was solved within 2 min; 0.5 when the solution was incomplete (i.e., all the dots except for the final one) at the expiry of the 2 min; 0 otherwise.
(2) *Planning time*: the response latency, in seconds, from the time the child receives the instructions until when s/he starts tracing the path on the grid.

The ToL assesses problem-solving and planning skills in children and adolescents (Luciana et al., 2009). The version used in this study is standardized for a population aged 4–13 years (Fancello et al., 2013). The task requires reproducing a configuration of three colored balls (blue, red, and green) on three vertical sticks of different heights, according to a set of rules: moving one ball at a time; once picked up, not holding the ball or placing it on the table; not placing more than one ball on the lower stick; not placing more than two balls on the medium stick. The entire test consists of 12 trials of increasing difficulty. Only one attempt per trial was allowed, and all 12 trials were presented, with no interruption criteria. The children’s performance was scored for:

(1) *Accuracy*: the attempt was scored 1 if the child performed the trial correctly within 1 min, without breaking any rule; 0 otherwise.
(2) *Planning time*: the seconds elapsed from when the trial is shown to the child until when s/he makes the first move.

##### Response inhibition skills

The *inhibition (squares/circles) subtest* of NEPSY-II (Korkman et al., 2007) and the *Numerical Stroop test* of the *Batteria Italiana ADHD* (BIA, Marzocchi et al., 2010) were used to assess children’s ability to inhibit automatic responses.

The *NEPSY-II inhibition (squares/circles) subtest* is standardized for children aged 3–16 (Korkman et al., 2007). The child is presented with a sheet displaying a set of figures (squares and circles) in five rows (eight figures per row) and asked to name aloud the figures from left to right as quickly and accurately as possible. The *inhibition task* is then performed: the child is instructed to say “circle” when seeing a square, and say “square” when seeing a circle, thus inhibiting automatic name retrieval. The children’s execution time is recorded.

The children’s performance was scored for:

(1) *Accuracy*: number of errors and self-corrections made by the child in performing the task;
(2) *Inhibition time*: the seconds required to complete the task.

The *Numerical Stroop test* of the BIA (Marzocchi et al., 2010) is standardized for children aged 6–11. The test assesses response inhibition by asking the child to suppress automatic digits recognition to pronounce the number of digits (ranging from 1 to 5) displayed on a table. Each cell of the table shows a digit from 1 to 5 repeated *n* times (for example, the digit 5, repeated three times). The child is asked to say as quickly and accurately as possible how many times the given digit (in the example, “5”) is shown in the cell (in the example, “three” times). The children’s performance is scored for:

(1) *Accuracy*: number of errors and self-corrections;
(2) *Inhibition time*: the seconds required to complete the task.

#### Data Analyses

Scores distribution was checked by inspecting skewness and kurtosis. Four outliers were identified (two with absolute skewness >2 and two with absolute kurtosis >7) and deleted from subsequent analyses resulting in a final sample size of 76 (*n* = 42 for the training group and *n* = 34 for the waiting-list group). Levene tests showed that variance was homogeneous between groups. The first research question of this study was whether training coding skills through Code.org would enhance the planning and response inhibition skills of first graders. Our hypothesis was that learning to code would enhance not only children’s coding skills but also their planning and response inhibition, increasing planning time and accuracy on standardized planning tasks, and contributing to decrease inhibition errors and inhibition time on standardized inhibition tasks. The second research question of the study was whether the positive effects of such training would be retained at 1 month from the end of the intervention. We predicted that positive training effects would be maintained.

As assignment to the different treatment conditions was at classroom level, a multilevel analysis was initially conducted to test the hypotheses of the study, while accounting also for the nested structure of the data. Intervention effects were tested by comparing the post-test performance of the two groups, with classroom as random contextual factor. Age, SES, and pre-test scores were included as covariates. The models showed non-significant and insufficient inter-cluster variance (across classrooms). Only intra-cluster variance (i.e., at participant level) was significant. As only the fixed-factor (group) and the covariates accounted for significant variance in children’s performance scores, analyses of variance (ANOVAs) were subsequently used to test the effects of the intervention and their maintenance. According to our hypotheses, learning to code (i.e., improvements in coding skills) would transfer to planning and response inhibition skills. Thus, we first tested that the training was effective in developing coding skills, and then verified its effects on children’s planning and response inhibition skills. Accordingly, planning time and accuracy on the coding tasks, planning time and accuracy on the Elithorn and ToL tasks, and inhibition time and accuracy on the NEPPSY-II and the numerical Stroop task were the dependent measures of the ANOVAs. A two (Group: experimental, waiting-list control) × two (Time: T2-post-test, T3-delayed post-test) mixed ANOVA tested the effects of the intervention. SES, age, and pre-test scores were covaried. Pre-test (T1) scores were covaried to control for variance in the dependent variables at the pre-test. This analytic strategy allowed testing in the same analysis both hypothesis 1 (the positive effects of the coding training) and hypothesis 2 (retention of the training effects at the delayed post-test). As the experimental group received the intervention between T1 and T2, while the wait list control group received it between T2 and T3 (see Figure 1), an interaction between Group and Time was expected, with better performance of the experimental group at the post-test (T2) and significant improvement of the performance of the wait list control group only between T2 and T3. Lack of significant differences between T2 (post-test) and T3 (delayed post-test) for the experimental group would indicate that the training effects were retained at 1 month from the end of the intervention. Significant interactions were explored by paired- and independent-samples *t*-tests. Effect sizes were computed using Cohen’s *d*, and correlations between repeated measures were used to correct for dependence between means (Morris and DeShon, 2002).

### Results

Between-group differences in age and SES and in the dependent (EF and coding) variables’ pre-test scores were explored by *t*-tests. A chi-square analysis was conducted to test for differences in gender distribution. The analyses showed that the two groups were equivalent for age, *t*(74) = −0.63, *p* = 0.53, SES, *t*(74) = −1.21, *p* = 0.23, and gender, χ*^2^* = 1.39, *p* = 0.24. Statistically significant differences between the groups at the pre-test were found for accuracy on the coding task, *t*(74) = −3.47, *p* = 0.001 and the ToL, *t*(74) = −2.88, *p* = 0.005. In both cases, the experimental group showed a better pre-test performance than the wait list control group (see Tables 3, 4). The difference approached significance for inhibition time and errors on the NEPSY-II, *t*(74) = 2.00, *p* = 0.05 and *t*(74) = 1.96, *p* = 0.05 (see Table 3). In the following, we report the results of the mixed ANOVAs for each dependent measure (planning time and accuracy at coding tasks, and planning time and accuracy, response inhibition time, and errors at standardized tasks).

**TABLE 3:** Study 1—between-group comparison: planning and response inhibition at T1, T2, and T3.

		**Wait list**	**Experimental**	**Independent**	**Cohen’s *d***
		***M* (*SD*)**	***M* (*SD*)**	**samples *t*-test**	
Planning time Elithorn	T1^pre–test^	22.76 (15.97)	25.71 (13.92)	–0.859	0.19
	T2^post–test^	20.61 (11.61)	25.50 (10.38)	–1.93	0.45
	T3^delayed post^	23.88 (9.59)	23.18 (7.78)	0.35	–0.08
Accuracy Elithorn	T1^pre–test^	5.47 (3.35)	6.75 (2.87)	–1.79	0.41
	T2^post–test^	7.29 (3.53)	9.96 (3.04)	–3.54^∗∗∗^	0.82
	T3^delayed post^	11.06 (3.29)	11.51 (2.54)	–0.677	0.15
Planning time ToL	T1^pre–test^	9.20 (4.42)	7.77 (3.33)	1.60	–0.37
	T2^post–test^	8.21 (3.33)	7.22 (3.09)	1.35	–0.31
	T3^delayed post^	9.04 (4.17)	7.93 (3.77)	1.21	–0.28
Accuracy ToL	T1^pre–test^	6.03 (2.47)	7.52 (2.05)	–2.88^∗∗^	0.66
	T2^post–test^	7.85 (2.08)	9.71 (1.86)	–4.11^∗∗∗^	0.95
	T3^delayed post^	10.29 (2.29)	9.93 (1.99)	0.74	–0.17
Inhibition time NEPSY-II	T1^pre–test^	56.21 (14.57)	50.97 (7.83)	2.00	–0.46
	T2^post–test^	47.88 (11.46)	45.45 (7.92)	1.09	–0.25
	T3^delayed post^	39.66 (9.21)	42.33 (8.24)	–1.33	0.31
Errors NEPSY-II	T1^pre–test^	3.56 (2.58)	2.43 (2.43)	1.96	–0.45
	T2^post–test^	2.85 (2.35)	1.56 (1.65)	2.84^∗^	–0.65
	T3^delayed post^	1.26 (1.78)	2.02 (2.36)	–1.55	0.36
Inhibition time Stroop	T1^pre–test^	216.3 (65.93)	218.0 (56.13)	–0.12	0.03
	T2^post–test^	186.1 (69.85)	178.1 (36.75)	0.64	–0.15
	T3^delayed post^	152.3 (37.11)	157.2 (39.20)	–0.56	0.13
Errors Stroop	T1^pre–test^	7.97 (6.14)	6.83 (6.47)	0.78	–0.18
	T2^post–test^	5.47 (5.09)	2.02 (2.38)	3.89^∗∗∗^	–0.90
	T3^delayed post^	2.53 (2.38)	3.33 (3.91)	–1.05	0.24

*^∗∗∗^*p* ≤ 0.001; ^∗∗^*p* < 0.005; ^∗^*p* < 0.01. Adjusted *p* = 0.02 after Bonferroni corrections.*

**TABLE 4:** Study 1—between-group comparison: performance at the coding tasks at T1, T2, and T3.

		**Wait list**	**Experimental**	**Independent**	**Cohen’s *d***
		***M* (*SD*)**	***M* (*SD*)**	**samples *t*-test**	
Planning time coding	T1^pre–test^	48.00 (23.29)	42.30 (22.45)	1.08	–0.25
	T2^post–test^	38.95 (25.26)	11.56 (6.29)	6.78^∗∗∗^	–1.56
	T3^delayed post^	11.33 (6.89)	11.54 (4.43)	–0.16	0.04
Accuracy coding	T1^pre–test^	3.09 (1.60)	4.31 (1.46)	–3.47^∗∗∗^	0.80
	T2^post–test^	3.68 (1.92)	6.12 (1.06)	–7.03^∗∗∗^	1.62
	T3^delayed post^	5.70 (0.94)	5.81 (1.09)	–0.44	0.11

*^∗∗∗^*p* < 0.001. Adjusted *p* = 0.02 after Bonferroni corrections.*

#### Effects of Learning to Code on Coding Skills: Planning Time

The covariates planning time at T1 and age were significant: *F*(1,71) = 6.49, *p* = 0.01, $\eta_{p}^{2}$ = 0.08, and *F*(1,71) = 4.42, *p* = 0.05, $\eta_{p}^{2}$ = 0.06. The main factor Group was also significant, with a large effect size: *F*(1,71) = 36.04, *p* < 0.001, $\eta_{p}^{2}$ = 0.34. Finally, also the interaction between Time and Group was significant (the effect size was very large): *F*(1,71) = 46.56, *p* < 0.001, $\eta_{p}^{2}$ = 0.40. At the post-test (T2), the experimental group spent significantly less time than the waiting-list (control) group on planning, *t*(74) = 6.78, *p* < 0.001, Cohen’s *d* = −1.56 (the effect size was very large), but no significant differences between the two groups were observed at T3, after the wait list control group received the intervention, *t*(74) = −0.16, *p* = 0.87 (see also Table 4). Between T2 and T3, the waiting-list group’s planning time decreased significantly, with a very large effect size, *t*(33) = −6.53, *p* < 0.001, Cohen’s *d* = 3.21, whereas no significant differences were observed for the experimental group, *t*(41) = −0.022, *p* = 0.98.

#### Effects of Learning to Code on Coding Skills: Accuracy

The covariates coding pre-test accuracy and age were significant, respectively: *F*(1,71) = 31.72, *p* < 0.001, $\eta_{p}^{2}$ = 0.31, and *F*(1,71) = 11.96, *p* = 0.001, $\eta_{p}^{2}$ = 0.14. Group was significant, *F*(1,71) = 13.00, *p* = 0.001, $\eta_{p}^{2}$ = 0.15. The effect size was large. Moreover, also the interaction Time × Group was significant, with a very large effect size: *F*(1,71) = 32.93, *p* < 0.001, $\eta_{p}^{2}$ = 0.32. Table 4 shows that the experimental group, who received the coding intervention between T1 and T2, performed significantly better than the wait list control group at the post-test (T2): *t*(74) = −7.03, *p* < 0.001, Cohen’s *d* = 1.62 (the effect size was very large). However, at T3, once the waiting-list group was exposed to the intervention, the difference between the two groups was no longer significant, *t*(74) = −0.44, *p* = 0.66. In fact, the performance of the waiting-list group improved significantly between T2 and T3, with the intervention, *t*(33) = 6.63, *p* < 0.001, Cohen’s *d* = −1.94 (the effect size was very large), whereas that of the experimental group remained stable, *t*(41) = −1.73, *p* = 0.09.

#### Effects of Learning to Code on Planning Skills: Planning Time

##### Elithorn

The ANOVA did not reveal significant effects of Group or Time on Elithorn planning time. The covariates (age, SES, and pre-test Elithorn planning time) were non-significant.

##### ToL

The covariates, pre-test planning time, and age were significant: respectively, *F*(1,71) = 17.16, *p* < 0.001, $\eta_{p}^{2}$ = 0.19 and *F*(1,71) = 8.94, *p* < 0.005, $\eta_{p}^{2}$ = 0.11. The main factor Time was also significant, *F*(1,71) = 4.44, *p* < 0.05, $\eta_{p}^{2}$ = 0.06. The means reported in Table 3 show that planning time slightly increased for both groups between T2 and T3. Group and the interaction Time × Group were non-significant.

#### Effects of Learning to Code on Planning Skills: Planning Accuracy

##### Elithorn

**FIGURE 2:**
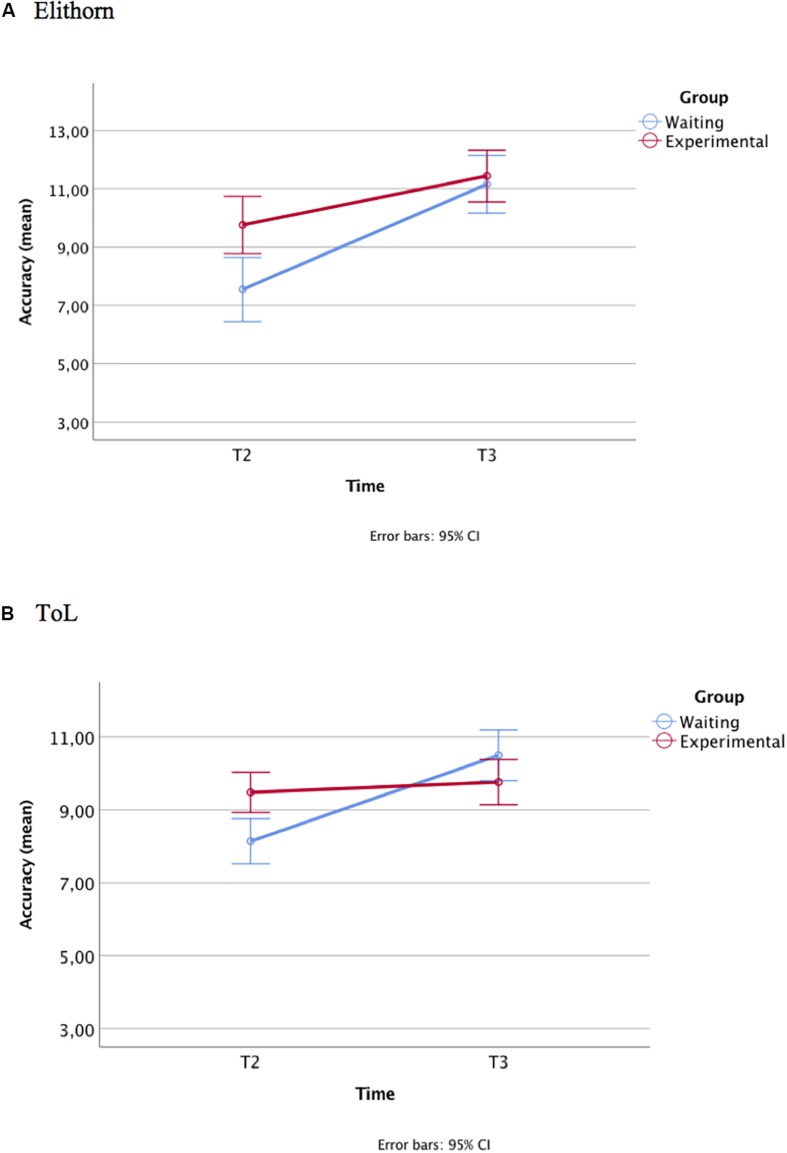
Study 1: planning accuracy at T2 and T3 (age, SES, and accuracy at Tl covariates) at the Elithorn **(A)** and ToL **(B)** tasks.

The covariate Elithorn pre-test accuracy was significant, *F*(1,71) = 9.65, *p* < 0.005, $\eta_{p}^{2}$ = 0.12. Group and the interaction Time × Group were also significant, both with a medium effect size: *F*(1,71) = 4.62, *p* < 0.05, $\eta_{p}^{2}$ = 0.06 (Group), and *F*(1,71) = 5.28, *p* < 0.05, $\eta_{p}^{2}$ = 0.07 (Time × Group). The *post hoc t*-tests, reported in Table 3, show that at the post-test (T2) the experimental group performed significantly better than the control group: *t*(74) = −3.54, *p* < 0.001, Cohen’s *d* = 0.80. The effect size was large. However, at the delayed post-test (T3), the wait list control group caught up with the experimental group: *t*(74) = −0.677, *p* = 0.500. The paired-samples *t*-tests showed that the waiting-list group improved indeed significantly from T2 to T3 (the effect size was large): *t*(33) = 5.68, *p* < 0.001, Cohen’s *d* = −1.01. Also, the experimental group improved, but less: *t*(41) = 3.19, *p* < 0.005, Cohen’s *d* = 0.55 (see Figure 2A).

##### ToL

The covariates age and pre-test ToL accuracy were significant, respectively, *F*(1,71) = 7.01, *p* = 0.01, and $\eta_{p}^{2}$ = 0.09, and *F*(1,71) = 18.10, *p* < 0.001, and $\eta_{p}^{2}$ = 0.20. The interaction Time × Group was significant, *F*(1,71) = 16.84, *p* < 0.001, and $\eta_{p}^{2}$ = 0.19. The effect size of the interaction was large. The experimental group performed significantly better than the wait list control group at T2 (the post-test), *t*(74) = −4.11, *p* < 0.001, Cohen’s *d* = 0.95. Between T2 and T3, with the intervention, the performance of the waiting-list group improved significantly: *t*(33) = 6.30, *p* < 0.001, *d* = −1.03 (the effect size was large), equaling that of the experimental group at T3, *t*(74) = 0.744, *p* = 0.459 (see Table 3). No significant differences were found between T2 and T3 for the experimental group, *t*(41) = −0.795, *p* = 0.43, indicating that the performance of this group remained stable (see Figure 2B).

#### Effects of Learning to Code on Response Inhibition Skills: Response Inhibition Time

##### NEPSY-II

The covariate, pre-test inhibition time, and the factor Group were significant, respectively: *F*(1,71) = 72.07, *p* < 0.001, $\eta_{p}^{2}$ = 50, and *F*(1,71) = 4.36, *p* < 0.05, $\eta_{p}^{2}$ = 06. The interaction Time × Group approached statistical significance, *F*(1,71) = 3.92, *p* = 0.05, $\eta_{p}^{2}$ = 0.05 (the effect size was medium). However, no significant differences emerged between the two groups at the post-test (T2): *t*(74) = 1.09, *p* = 0.28, or at the delayed post-test (T3), *t*(74) = −1.33, *p* = 0.19. Between the post-test (T2) and the delayed post-test (T3), inhibition time decreased significantly for both groups, with a large effect size for the waiting-list control group, *t*(33) = −4.68, *p* < 0.001, and, *d* = 0.92, and a small effect size for the experimental group, *t*(41) = −2.47, *p* < 0.05, and *d* = 0.37. Inspection of the means reported in Table 3 shows that the decrease in inhibition time was steady from T1 to T3 for both groups.

##### Stroop

The analyses revealed only an effect of the covariate, pre-test Stroop time, *F*(1,71) = 88.99, *p* < 0.001, $\eta_{p}^{2}$ = 0.56. Table 3 shows that the between-group difference was not significant at the post-test (T2), *t*(74) = 0.64, *p* = 0.52 or at the delayed post-test (T3), *t*(74) = −56, *p* = 0.58. For both groups, Stroop time decreased significantly between T2 and T3: The effect size was large for the wait list control group, *t*(33) = −3.62, *p* = 0.001, *d* = 1.07, and medium for the experimental group, *t*(41) = −4.29, *p* < 0.001, *d* = 0.64. Similar to the NEPSY-II inhibition task, a steady decrease in inhibition time from T1 to T3 was observed (see Table 3).

#### Effects of Learning to Code on Response Inhibition Skills: Response Inhibition Errors

##### NEPSY-II

**FIGURE 3:**
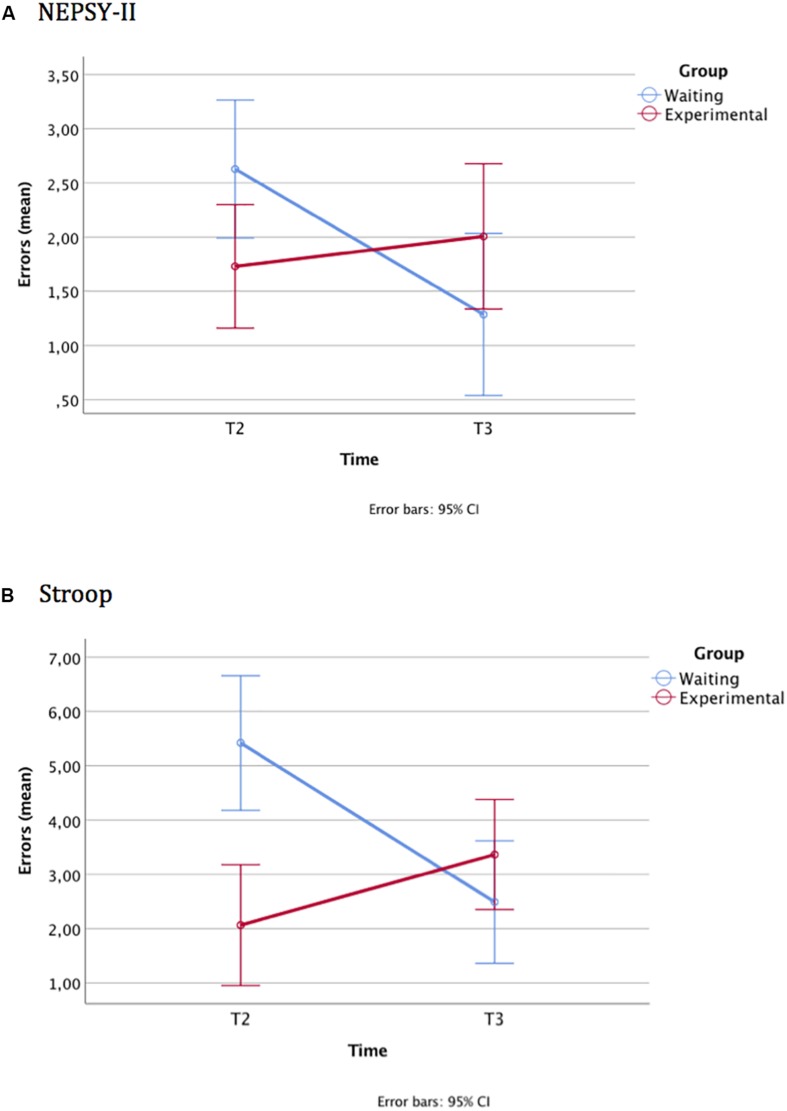
Study 1: errors in response inhibition at T2 and T3 (errors at Tl covariate): NEPSY-II **(A)** and Stroop **(B)** tasks.

The covariate, pre-test inhibition errors, was statistically significant, *F*(1,71) = 5.71, *p* < 0.05, $\eta_{p}^{2}$ = 0.07. The interaction Time × Group was also significant, *F*(1,71) = 7.97, *p* < 0.01, $\eta_{p}^{2}$ = 0.10 (the effect size was medium). The experimental group, who received the intervention between T1 and T2, made significantly fewer errors than the waiting-list group at T2, *t*(74) = 2.84, *p* < 0.01, Cohen’s *d* = −0.65 (the effect size was medium), but at T3, the performance of the two groups was equivalent, *t*(74) = −1.55, *p* = 0.12. Indeed, between T2 and T3, the wait list control group showed a significant decrease in the number of inhibition errors, *t*(33) = −3.76, *p* < 0.001, Cohen’s *d* = 0.76 (the effect size was medium). The performance of the experimental group remained instead stable in this time interval, *t*(41) = 1.18, *p* = 0.246 (Figure 3A).

##### Stroop

The covariate T1 Stroop errors was significant, *F*(1,71) = 11.76, *p* = 0.001, $\eta_{p}^{2}$ = 0.14. The interaction Time × Group was also significant, *F*(1,71) = 21.00, *p* < 0.001, $\eta_{p}^{2}$ = 0.23 and the effect size was very large. At T2, the experimental group made significantly fewer Stroop errors than the wait list control group, *t*(74) = 3.89, *p* < 0.001, Cohen’s *d* = −0.90 (the effect size was large). At T3, the difference between the two groups was no more significant, *t*(74) = −1.05, *p* = 0.30 (see Table 3), due to the significant decrease in the number of inhibition errors of the waiting-list group between T2 and T3, *t*(33) = −3.74, *p* = 0.001, Cohen’s *d* = 1.16 (see Figure 3B). The effect size was large. The number of Stroop errors slightly increased for the experimental group between T2 and T3, *t*(41) = 2.58, *p* = 0.01, Cohen’s *d* = −0.35. The effect size was small.

### Conclusions From Study 1

The results of study 1 confirmed that learning to code may benefit planning and response inhibition skills significantly even after relatively short practice with coding. A stepped-wedge cluster randomized trial design (Campbell et al., 2019) was used to test the effects of the intervention, with the experimental and wait list control group receiving the intervention at different times (the former between T1 and T2; the latter between T2 and T3). After the coding training, at T2, the experimental group outperformed the wait list control group on the two standardized planning tasks (Elithorn and ToL) and the two standardized inhibition tasks (NEPPSY-II and Stroop). Between T2 and T3, with the coding training, also the waiting-list control group improved significantly in coding and, with it, in planning and response inhibition, showing at T3 levels of performance equivalent to those of the experimental group. The performance of the experimental group remained stable, indicating that the positive effects of the coding training were retained at the delayed post-test. The only exception is the Stroop task, for which the performance of experimental group worsened between T2 and T3.

The benefits of the coding activities were also more evident on accuracy than on planning or inhibition time. In fact, the findings did not confirm the predicted increase of time spent planning following the intervention. A possible explanation of this unexpected effect is that the latency time before initiating the task (our planning time measure) may reflect other processes than planning alone (for example, children’s exploration of the problem space or familiarity with the task). Consistently with this interpretation, after the coding intervention, by becoming familiar with the Code.org platform and its tooling (e.g., the visual block commands), the children likely needed significantly less time to explore the visual interface and the trials. Consequently, their planning time (measured as response latency) decreased (rather than increase) and such decrease was associated with an increase in accuracy on the same tasks. (We return to this point below). Thus, this finding can be interpreted as an indication of the acquired efficiency of the children in solving the coding problems.

The analysis of performance on the coding and standardized tasks proves that the children exposed to coding not only learned to code, but also developed planning and response inhibition skills, showing significant transfer effects. To check whether the improvement observed in EF was associated to children’s gains in coding, bivariate correlations were run between change scores (i.e., score difference between T2 and T1 and between T3 and T2) in coding and the corresponding change scores in planning accuracy and response inhibition at the EF assessment. These further analyses showed that a decrease in planning time on the coding tasks between T1 and T2 was significantly associated with coding accuracy, *r*(76) = −0.61, *p* < 0.001, and with improvements in accuracy on the Elithorn and ToL tasks between T1 and T2, respectively, *r*(76) = −0.29, *p* = 0.01 and *r*(76) = −0.31, *p* < 0.01. Change scores in coding accuracy between T1 and T2 were also positively associated with change scores in accuracy on the Elithorn, *r*(76) = 0.26, *p* < 0.05. A decrease in planning time on the coding tasks, between T2 and T3, was significantly associated with change scores (improvement) in coding accuracy in the same time period, *r*(76) = −0.70, *p* < 0.001, with the improvement in accuracy on the Elithorn and ToL tests: *r*(76) = −0.38, *p* = 0.001 and *r*(76) = −0.47, *p* < 0.001, and also with a decrease in inhibition errors on the NEPSY-II, *r*(76) = 0.23, *p* < 0.05, and Stroop tasks, *r*(76) = 0.45, *p* < 0.001. Finally, improvements in coding accuracy between T2 and T3 were positively associated with improvements in accuracy on the Elithorn, *r*(76) = 0.33, *p* < 0.005, and ToL task, *r*(76) = 0.42, *p* < 0.001, and were negatively associated with the decrease in inhibition errors on the Stroop test, *r*(76) = −0.35, *p* < 0.005.

Complementing other recent investigations (Di Lieto et al., 2017) showing that experience with coding in tangible (i.e., physical) environments can improve significantly children’s working memory and inhibition skills, the findings of study 1 suggest that guided exposure to coding through a virtual learning environment can benefit considerably also more complex EFs such as planning, and these effects can be detected from an early age (5–6 years).

The question of whether learning to code can accelerate the development of 5–6-year-old children’s EFs significantly was further explored in study 2, by integrating these results with longitudinal data.

## Study 2

This second study explored further the effects of coding on children’s EFs by combining a longitudinal and randomized controlled trial design. The aims of the study were:

(1) To replicate the findings of study 1 with a group of second graders, novice to coding;
(2) To examine the extent to which coding experience could boost the spontaneous development of children’s planning and inhibition skills. We explored whether children’s improvements in planning and response inhibition following 1-month coding intervention were greater than those occurring in the same children in 7 months of spontaneous development and standard curricular activities.

**FIGURE 4:**
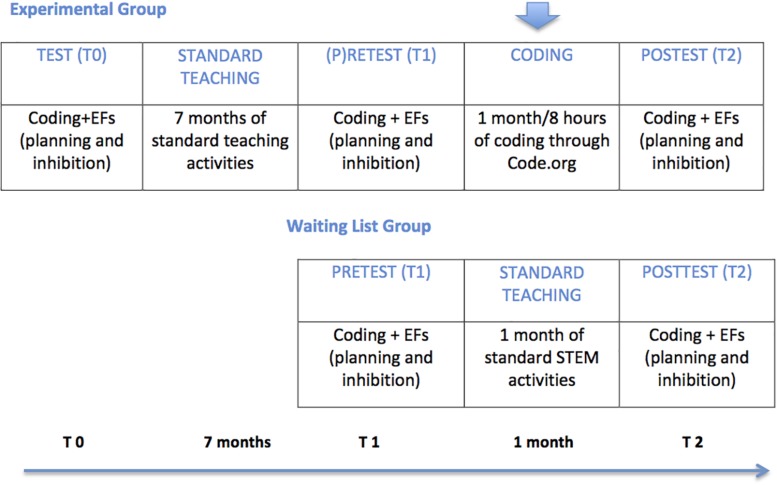
Experimental design study 2.

This experimental design was similar to that of study 1, except that one group of children (experimental group) was followed longitudinally, and tested at three time points (T0, test; T1, pre-test, after 7 months from T0, to assess the spontaneous development of EFs in a long time period; and at T2, post-test, after 1 month of exposure to coding). The other group (control group) was tested only twice (at T1 and T2) (see Figure 4).

### Participants

Thirty-eight second graders participated in this trial. The experimental group included 19 children followed longitudinally for 1 year, from grade 1 to grade 2 (7 girls, 37%, 12 boys, 63.2%, mean age, 6.89), the control group consisted of other 19 second graders matched on age, gender, and SES to the experimental group (10 girls, 53%, 9 boys, 47.4%, mean age 6.89), from a different school. All children were native speakers of Italian and not signaled for learning disabilities or other developmental disorders. Parental written informed consent was collected before the study for all participants. Demographic data are reported in Table 5.

**TABLE 5:** Study 2—demographic characteristics of the experimental and control group.

	**Experimental**	**Control**	***p*-value**
**Gender**			
Girls (*n*, %)	7, 36.8%	10, 52.6%	0.32
Boys (*n*, %)	12, 63.2%	9, 47.4%	
Age (*M*, *SD*)	6.89 (0.205)	6.89 (0.315)	1.00
SES (*M*, *SD*)	6.11 (1.56)	6.79 (1.18)	0.14

### Procedure and Materials

The procedure and materials were the same as for study 1.

Results are presented separately for the randomized controlled trial and longitudinal part of the study.

### Results of the Randomized Controlled Trial

The two groups were equivalent in Age, *t* < 1, *p* = 1.00, SES, *t*(36) = −1.52, *p* = 0.14, and for gender distribution, *χ*^2^ = 0.96, *p* = 0.32. Between-group differences at the pre-test (T1) were explored by *t*-tests, which confirmed that the two groups did not differ significantly in any dependent measure except for T1 accuracy on the ToL, *t*(36) = 2.22, *p* = 0.03, where the control group outperformed the experimental group.

Skewness and kurtosis values were within critical thresholds, with the exception of Stroop time T1, for which kurtosis slightly exceeded the critical value of 3.00 (kurtosis = 3.54). Levene tests confirmed equal variance between the two groups. Between-group ANOVAs were thus used to address the first objective of the study (i.e., replicate the results of study 1 with second graders) and explore between-group differences in the dependent measures at T2 (post-test) with T1 (pre-test) performance, age, and SES as covariates. Table 6 displays group means and independent samples *t*-tests for group comparison at the two time points (T1 and T2). Similar to study 1, the intervention effects on children’s coding skills were tested first, followed by transfer effects on children’s planning and response inhibition.

**TABLE 6:** Study 2—between-group comparison: planning and response inhibition at T1 (pre-test) and T2 (post-test).

		**Control**	**Experimental**	**Independent**	**Cohen’s *d***
		***M* (*SD*)**	***M* (*SD*)**	**samples *t*-test**	
Planning time Elithorn	T1^pre–test^	24.34 (11.72)	20.27 (11.58)	1.09	–0.35
	T2^post–test^	18.24 (8.41)	19.17 (8.26)	–0.34	0.11
Accuracy Elithorn	T1^pre–test^	9.26 (4.19)	9.79 (4.91)	–0.36	0.12
	T2^post–test^	9.00 (4.10)	12.68 (3.33)	–3.04^∗∗^	0.96
Planning time ToL	T1^pre–test^	5.48 (2.64)	5.34 (2.14)	0.19	–0.06
	T2^post–test^	4.77 (2.14)	6.52 (3.15)	−2.00^#^	0.65
Accuracy ToL	T1^pre–test^	8.58 (2.27)	7.00 (2.11)	2.22^#^	–0.72
	T2^post–test^	8.11 (2.49)	10.16 (1.86)	−2.87^∗^	0.93
Inhibition time NEPSY-II	T1^pre–test^	36.88 (7.26)	35.75 (8.39)	0.44	–0.14
	T2^post–test^	37.51 (7.22)	34.05 (9.77)	1.24	–0.40
Errors NEPSY-II	T1^pre–test^	3.79 (2.68)	3.74 (3.31)	0.05	–0.02
	T2^post–test^	2.89 (2.13)	1.05 (1.27)	3.24^∗∗^	–1.05
Inhibition time Stroop	T1^pre–test^	124.88 (14.72)	138.24 (26.62)	–1.91	0.62
	T2^post–test^	127.77 (16.58)	132.27 (30.80)	–0.56	0.18
Errors Stroop	T1^pre–test^	3.68 (2.89)	4.32 (4.29)	–0.53	0.17
	T2^post–test^	2.74 (2.42)	2.11 (2.35)	0.82	–0.26

*^∗∗^*p* < 0.005; ^∗^*p* < 0.01, ^#^*p* ≤ 0.05. Adjusted *p* = 0.02 after Bonferroni corrections.*

#### Effects of Learning to Code on Coding Skills: Planning Time

The covariate, pre-test planning time was significant, *F*(1,33) = 19.60, *p* < 0.001, $\eta_{p}^{2}$ = 0.37, and no significant effects of Group were observed. As shown in Table 7, the two groups spent equivalent time planning both at T1 and T2.

**TABLE 7:** Study 2—between-group comparison: performance at coding tasks at T1 (pre-test) and T2 (post-test).

		**Control**	**Experimental**	**Independent**	**Cohen’s *d***
		***M* (*SD*)**	***M* (*SD*)**	**samples *t*-test**	
Planning	T1^pre–test^	9.77 (3.62)	7.42 (4.36)	1.81	–0.59
time Coding	T2^post–test^	8.46 (2.47)	7.78 (3.80)	0.65	–0.21
Accuracy	T1^pre–test^	5.58 (1.17)	6.05 (1.08)	–1.30	0.42
Coding	T2^post–test^	5.21 (1.08)	7.16 (0.96)	–5.87^∗∗∗^	1.91

*^∗∗∗^*p* < 0.001. Adjusted *p* = 0.02 after Bonferroni corrections.*

#### Effects of Learning to Code on Coding Skills: Accuracy

The analyses revealed a significant effect of the covariate T1 coding accuracy, *F*(1,33) = 25.95, *p* < 0.001, $\eta_{p}^{2}$ = 0.44, and of Group, *F*(1,33) = 38.11, *p* < 0.001, $\eta_{p}^{2}$ = 0.54. (The effect size was very large). Table 7 shows that whereas at T1, the performance of the two groups was equivalent, at T2, the experimental group performed significantly better than the control group, and the effect size was very large: *t*(36) = −5.87, *p* < 0.001, Cohen’s *d* = 1.91.

#### Effects of Learning to Code on Planning Skills: Planning Time

##### Elithorn

Only the covariates Age and planning time at T1 were significant: *F*(1,33) = 4.78, *p* < 0.05, $\eta_{p}^{2}$ = 0.13, and *F*(1,33) = 4.77, *p* < 0.05, $\eta_{p}^{2}$ = 0.13. Group was not significant. As shown in Table 6, the independent-samples *t*-tests did not reveal statistically significant differences between the two groups neither at T1 nor at T2.

##### ToL

The covariate T1 planning time was significant, *F*(1,33) = 30.61, *p* < 0.001, $\eta_{p}^{2}$ = 0.48. Group was statistically significant, *F*(1,33) = 11.04, *p* < 0.005, $\eta_{p}^{2}$ = 0.25, and the effect size was very large. At T1, the two groups spent equivalent time planning (see Table 6), whereas at the post-test (T2), the experimental group spent more time planning than the control, and the difference approached statistical significance once Bonferroni corrections were applied: *t*(36) = −2.00, *p* = 0.05, Cohen’s *d* = 0.65. The effect size was medium.

#### Effects of Learning to Code on Planning Skills: Planning Accuracy

##### Elithorn

The covariate T1 accuracy was statistically significant, *F*(1,33) = 35.06, *p* < 0.001, $\eta_{p}^{2}$ = 0.51. Group was also statistically significant, *F*(1,33) = 15.94, *p* < 0.001, $\eta_{p}^{2}$ = 0.32. The effect size was very large. As shown also in Table 6, at T1, the performance of the two groups was equivalent, whereas at the post-test (T2), the experimental group performed significantly better than the control group, with a large effect size: *t*(36) = −3.04, *p* < 0.005, Cohen’s *d* = 0.96.

##### ToL

Also for the ToL, the covariate T1 accuracy was significant, *F*(1,33) = 23.10, *p* < 0.001, $\eta_{p}^{2}$ = 0.41. The analysis showed a significant difference between the two groups at the post-test (T2): *F*(1,33) = 29.32, *p* < 0.001, $\eta_{p}^{2}$ = 0.47 (the partial eta-squared shows that the effect size was very large). Inspection of Table 6 shows that while the control group outperformed the experimental group at the pre-test (T1), *t*(36) = 2.22, *p* < 0.05, Cohen’s *d* = −0.72 (the effect size was medium), the situation reversed at the post-test (T2), where the experimental group performed significantly better, *t*(36) = −2.87, *p* < 0.01, Cohen’s *d* = 0.93. The effect size was large.

#### Effects of Learning to Code on Response Inhibition Skills: Response Inhibition Time

##### NEPSY-II

The analysis did not reveal any significant between-group difference. Only the covariate T1 inhibition time was statistically significant, *F*(1,33) = 69.43, *p* < 0.001, $\eta_{p}^{2}$ = 0.68.

##### Stroop

Like for the NEPSY-II inhibition task, only the covariate T1 Stroop time was significant, *F*(1,33) = 37.19, *p* < 0.001, $\eta_{p}^{2}$ = 0.53. The independent-samples *t*-tests showed a difference in inhibition time between the two groups, approaching significance at T1, *t*(36) = −1.91, *p* = 0.06, Cohen’s *d* = 0.62. The experimental group showed longer inhibition time than the control and the effect size was medium. Yet, the two groups did not differ significantly at the post-test (T2) (see Table 6).

#### Effects of Learning to Code on Response Inhibition Skills: Response Inhibition Errors

##### NEPSY-II

The covariate T1 inhibition errors were significant, *F*(1,33) = 14.63, *p* < 0.001, $\eta_{p}^{2}$ = 0.31. Group was also statistically significant, *F*(1,33) = 10.75, *p* < 0.005, $\eta_{p}^{2}$ = 0.25. The effect size was large. The independent-samples *t*-tests showed that the performance of the two groups did not differ significantly at the pre-test (T1) (see Table 6). However, at the post-test (T2), the experimental group made significantly fewer errors than the control group and the effect size was large: *t*(36) = 3.24, *p* < 0.005, Cohen’s *d* = −1.05.

##### Stroop

On the Stroop task, only the pre-test errors resulted significant, *F*(1,33) = 26.19, *p* < 0.001, $\eta_{p}^{2}$ = 0.44 (see Table 6). The performance of the two groups did not differ significantly at T1 or at T2.

Overall, the results of study 2 largely replicated those of study 1: the experimental group improved more than the control group in the ability to code, while greater gains in EFs (planning and response inhibition) were observed than those made by the control group. After the coding training, the experimental group spent significantly more time planning on the ToL and was significantly more accurate than the control group on both standardized planning tasks (Elithorn and ToL). The experimental group also made significantly fewer errors than the control group on the NEPSY-II inhibition task. Pearson correlations confirmed that change scores (between T1 and T2) in planning and response inhibition were significantly associated with change scores in coding accuracy and time planning on coding tasks. Like in study 1, change scores in coding accuracy and coding planning time were significantly correlated: *r*(38) = 0.46, *p* < 0.005. Yet, unlike study 1 (in which a negative correlation occurred between time spent planning and accuracy), a positive correlation emerged between these two measures: the increased accuracy on coding tasks was associated with increased time spent planning in the coding tasks and with increased time planning on the ToL, *r*(38) = 0.43, *p* < 0.01. Moreover, increase in planning time on the coding and on the ToL tasks were significantly correlated: *r*(38) = 0.35, *p* < 0.05. Positive significant correlations were also found between children’s gains in coding accuracy and gains in accuracy on the Elithorn, *r*(38) = 0.38, *p* < 0.05, and ToL, *r*(38) = 0.35, *p* < 0.05, tasks. Finally, increased accuracy on coding tasks was significantly associated with a decrease of errors in the NEPSY-II inhibition task.

The experimental group was followed longitudinally and tested also at T0, 7 months before the pre-test (T1) and the intervention. The longitudinal data refer to only 17 children, as two children of this group were not assessed at T0. To determine in which measure the coding intervention boosted the development of the children’s EFs (the second objective of study 2), we compared the changes in the EFs of the experimental group between T0 and T1, i.e., a period of 7 months in which they were *not* exposed to coding, to those occurring between T1 and T2, after 1 month (4 weeks) of coding training. Change scores were used to compare children’s improvement in EFs and coding between the T0–T1 and T1–T2 time intervals. Cohen’s *d* effect size was calculated and correlations between repeated measures were used to correct for dependence between means (Morris and DeShon, 2002). Means and standard deviations are reported in Table 8.

**TABLE 8:** Study 2–longitudinal data: performance of the experimental group at T0 (test), at T1 (pre-test), and at T2 (post-test).

	**Time**	**Score**	**Change**	**Paired**	**Cohen’s *d***
		***M* (*SD*)**	**score**	***t*-test**	
Planning time	T0	16.32 (12.66)			
Elithorn	T1^pre–test^	19.23 (11.69)	2.91^*T1*−*T0*^		
	T2^post–test^	17.47 (5.83)	−1.76^*T2*−*T1*^	0.78	–0.19
Accuracy	T0	5.65 (3.30)			
Elithorn	T1^pre–test^	8.91 (4.72)	3.26^*T1*−*T0*^		
	T2^post–test^	12.71 (3.50)	3.79^*T2*−*T1*^	–0.27	0.08
Planning time	T0	5.39 (1.33)			
ToL	T1^pre–test^	5.10 (2.14)	−0.29^*T1*−*T0*^		
	T2^post–test^	6.46 (3.30)	1.35^*T2*−*T1*^	–1.82	0.44
Accuracy ToL	T0	6.00 (1.87)			
	T1^pre–test^	7.12 (2.18)	1.12^*T1*−*T0*^		
	T2^post–test^	10.18 (1.98)	3.06^*T2*−*T1*^	−2.18^#^	0.62
Inhibition time	T0	36.44 (4.77)			
NEPSY-II	T1^pre–test^	34.13 (6.81)	−2.31^*T1*−*T0*^		
	T2^post–test^	31.89 (6.63)	−2.24^*T2*−*T1*^	–0.02	0.01
Errors	T0	2.12 (2.20)			
NEPSY-II	T1^pre–test^	3.76 (3.47)	1.64^*T1*−*T0*^		
	T2^post–test^	1.06 (1.30)	−2.70^*T2*−*T1*^	2.82^∗^	–0.74
Inhibition time	T0	157.68 (22.05)			
Stroop	T1^pre–test^	134.21 (21.29)	−23.47^*T1*−*T0*^		
	T2^post–test^	127.71 (25.16)	−6.49^*T2*−*T1*^	–1.70	–38
Errors Stroop	T0	7.00 (8.82)			
	T1^pre–test^	4.35 (4.50)	−2.65^*T1*−*T0*^		
	T2^post–test^	2.24 (2.44)	−2.12^*T2*−*T1*^	–0.18	0.03
Planning time	T0	13.00 (5.24)			
Coding	T1^pre–test^	6.70 (2.54)	−6.30^*T1*−*T0*^		
	T2^post–test^	7.15 (3.18)	0.45^*T2*−*T1*^	–3.58^∗∗^	0.75
Accuracy	T0	4.29 (0.920)			
Coding	T1^pre–test^	6.06 (1.14)	1.76^*T1*−*T0*^		
	T2^post–test^	7.24 (0.970)	1.18^*T2*−*T1*^	1.11	–0.24

*^∗∗^*p* < 0.005; ^∗^*p* < 0.01; ^#^*p* ≤ 0.05. Adjusted *p* = 0.02 after Bonferroni corrections.*

### Longitudinal Data: Results

#### Effects of Learning to Code on Coding Skills: Planning Time

The difference between the two time intervals (T0–T1 and T1–T2) was significant *t*(16) = −3.58, *p* < 0.005, Cohen’s *d* = 0.75. The (negative) change score between T0 and T1, indicating a decrease in the time spent planning, was larger than the (positive) change score (increase in planning time) between T1 and T2 (see Table 8).

#### Effects of Learning to Code on Coding Skills: Accuracy

Accuracy in the coding tasks increased from T0 to T1 and from T1 to T2. The dimension of the change was not significantly different between the two time intervals.

#### Effects of Learning to Code on Planning Skills: Planning Time

##### Elithorn

No statistically significant difference was found between the two time intervals.

##### ToL

Also for the ToL, no statistically significant difference was found. The means reported in Table 8 show that the time spent planning on the ToL decreased between T0 and T1 and increased between T1 and T2, but the difference between the change scores was not significant.

#### Effects of Learning to Code on Planning Skills: Planning Accuracy

##### Elithorn

On the Elithorn, the difference in the accuracy change scores between T0–T1 and T1–T2 was not significant. The means in Table 8 show an equivalent improvement in accuracy during the two time intervals.

##### ToL

**FIGURE 5:**
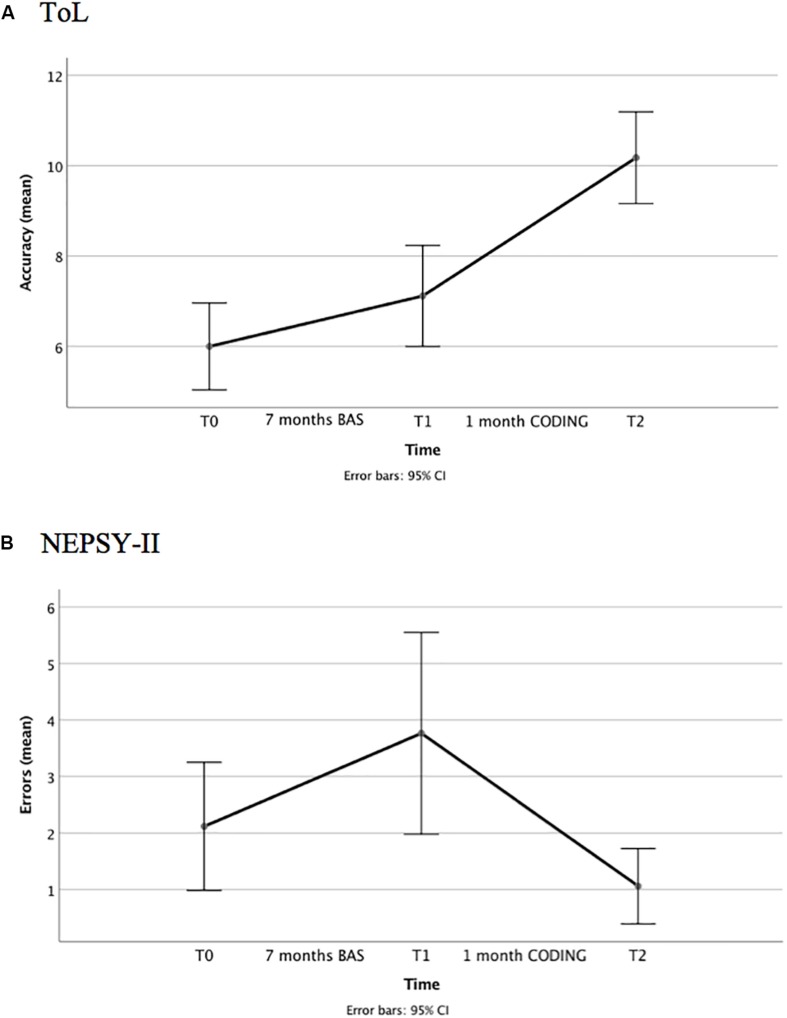
Study 2: longitudinal data: **(A)** ToL accuracy and **(B)** NEPSY-II inhibition errors at TO (test), Tl (pre-test), and T2 (post-test). BAS = business-as-usual.

Applying Bonferroni corrections, the difference in change scores approached statistical significance, *t*(16) = −2.18, *p* = 0.04, Cohen’s *d* = 0.62. The effect size was medium. As shown by Figure 5A, the improvement in accuracy was significantly greater between T1 and T2 (1 month of exposure to coding) than between T0 and T1 (7 months of regular learning activities).

#### Effects of Learning to Code on Response Inhibition Skills: Response Inhibition Time

##### NEPSY-II

No significant differences emerged for inhibition time between T0–T1 and T1–T2. As shown by change scores in Table 8, children’s inhibition time decreased progressively from T0 to T2.

##### Stroop

Like for the NEPSY-II task, no significant differences were found between the two time intervals (T0–T1 and T1–T2).

#### Effects of Learning to Code on Response Inhibition Skills: Response Inhibition Errors

##### NEPSY-II

A statistically significant difference in change scores was found between the two time intervals, *t*(16) = 2.82, *p* = 0.01, Cohen’s *d* = −0.74. The effect size was medium. As shown in Figure 5B, inhibition errors increased between T0 and T1, but decreased between T1 and T2 (see also change scores reported in Table 8). The dimension of the change was larger between T1 and T2.

##### Stroop

The negative change scores reported in Table 8 indicate a decrease in inhibition errors from T0 to T1 and from T1 to T2. The difference between these two time intervals was not significant.

### Conclusions From Study 2

Study 2 replicated the findings of study 1, but also furthered our comprehension of the effects of coding on children’s EFs, showing that learning to code can boost the development of children’s EFs. The evidence we collected shows that children with no prior experience of coding may benefit from a short (1-month) coding intervention in terms of planning and response inhibition. Notably, the longitudinal data showed that, on the Elithorn task, the gains in planning after 1 month of coding experience were equivalent to those obtained in the development of the same function with 7 months of exposure to standard curricular activities. On the ToL task, which involves a greater extent of problem-solving skills (Luciana et al., 2009), the observed gains, measured by change scores, were greater than those occurring after 7 months of standard learning activities. Much like in study 1, we noted in study 2 too that the effects of the intervention were more apparent for accuracy than for planning and response inhibition time. Remarkably, inhibition errors decreased in the experimental group, followed longitudinally, only *after* the coding intervention and the change occurring during the time interval between T1 and T2 (1 month) was greater than that between T0 and T1. This finding suggests that focused and targeted instructional problem-solving activities, like those involved in coding, help boost inhibition skills in children in their first years of schooling.

It could be argued that the greater gains made by the experimental group in planning and response inhibition after the training were due to the shorter time lag (1 month versus 7 months) between the repeated standardized tasks, which could have emphasized task familiarity effects. However, the finding that similar improvements did not occur in the control group suggests that the effects observed do relate to the specific benefits of the training more than to task familiarity.

In terms of planning time, the effects of the intervention were evident only on the ToL task. The children of the experimental group spent more time planning than at the post-test, and they planned better (with more accuracy) than the control group. Moreover, the change in planning accuracy on the ToL was significantly greater than that obtained after 7 months of standard learning activities. The fact that the effects on planning time were limited to the ToL might reflect the nature of the task, which is more complex (and thus likely more sensitive) than the Elithorn, where the child can visually explore the tracks in the maze.

The relationship found between planning time and accuracy differs in the two studies. Whereas in study 1 their association is negative, indicating that an increase in planning accuracy corresponds to a decrease in planning time, the opposite appears in study 2: An increase in accuracy in the coding tasks correlates with an increase in the time spent planning. Several variables could explain these divergent findings, including children’s characteristics, or the different emphasis teachers may put on planning skills in regular classroom activities. A difference between study 1 and 2 is, however, the older age of the participants in study 2. Older children could be more self-regulated and thus more prone to plan (Magi et al., 2016; Poutanen et al., 2016). A quick comparison between the average planning time of study 1 and study 2 (see Tables 3, 6) suggests, though, that this is not the case: The children of study 1 devoted on average the same, or more, time planning than those of study 2. Yet, the participants of study 2 showed on average greater accuracy on the planning tasks (Elithorn and ToL). It may be that these older children were simply more efficient in using planning to perform the tasks.

## General Discussion

The two studies presented in this paper explored the effects of coding, a learning activity recently introduced in the primary school curriculum, on first and second graders’ planning and response inhibition skills. Examining the role played by everyday curriculum-based learning activities on children’s EFs is essential to taking informed educational decisions. Examples of such decisions include determining at what age specific learning activities should be introduced or what kind of activities can be more fruitful at a given age for children’s cognitive development.

As discussed earlier in this paper, the studies that explore the effects of curricular activities on the development of children’s EFs are often challenged by the fact that it is difficult to find a control group at equal educational level, not bound to receive the target intervention (e.g., reading, writing, or math) at the same time (Baker et al., 2015). The recent introduction of coding instruction in primary school offers a “natural experiment” to developmental and educational psychologists. Since its integration in national curricula worldwide is not yet completed, comparisons between children who receive coding intervention and children who do not indeed are possible.

The two studies reported in this paper suggest the opportunity to introduce children early—at the beginning of primary school—to CT by means of guided exposure to coding. Faced with the challenge of coding problems, children seem to develop not only response inhibition skills (that is, command of prepotent responses), but also more complex EFs such as planning abilities. The positive effect of coding on children’s inhibition skills has been observed earlier (Di Lieto et al., 2017) and our findings provide further confirmatory evidence in this direction. Furthermore, the two studies reported in this paper also provide the first empirical evidence that learning coding early in school positively affects complex EFs, such as planning.

Response inhibition and planning support learning and humans’ problems solving (Hongwanishkul et al., 2005; Altemeier et al., 2006; Roebers et al., 2011; Crook and Evans, 2014; Liu et al., 2015; Blair, 2017; Purpura et al., 2017). Thus, improvements in these skills may have in turn strong impact on children’s academic success and everyday life (Crook and Evans, 2014; Blair and Raver, 2016; Blair, 2017).

In general, the coding intervention deployed in the two studies reported in this paper has been more effective for the development of children’s planning than inhibition skills. The finding that planning skills are plastic in first and second graders and can be boosted effectively by curricular activities like coding is an important finding, especially so, considering that planning involves also more basic EF processes, such as inhibition and working memory (Luciana et al., 2009).

However, whereas the planning abilities developed through coding in studies 1 and 2 transferred to both standardized planning tasks (the Elithorn and the ToL), the effects on inhibition skills seemed less robust and generalized. In study 1, the accuracy gained in the Stroop task was not retained at 1 month from the intervention, and in study 2, the positive effects of the training did not generalize to the Stroop task. This observation could relate to general lesser plasticity of inhibition processes or to specific training effects, that is, to factors related to the nature of the training tasks or the duration of the training. As noted above, response inhibition is involved in planning (Luciana et al., 2009). However, promoting response inhibition indirectly through planning may lead to less strong or robust effects than direct interventions targeting inhibition skills. Another explanation is that longer training might be required to consolidate gains in response inhibition skills. Response inhibition may be more vulnerable indeed to situational and external factors (e.g., tiredness, mood) than planning. The latter, in fact, is a more complex cognitive process, which may involve greater strategic control. The hypothesis that the reduced effects on inhibition can originate from the short duration of the intervention matches findings that suggest that longer trainings lead to significant positive effects on children’s response inhibition (Di Lieto et al., 2017) and other EFs (Kronenberger et al., 2011).

### Limitations

The short duration of the coding intervention and the lack of a long-term follow-up are the two main limitations of the present studies. Di Lieto et al. (2017), who found positive effects of a coding training on 5-year-old children’s response inhibition, employed a longer training than the one we had in studies 1 and 2: 13 sessions/6 weeks versus 8 sessions/4 weeks. Other EF trainings destined to children of similar age to those involved in these studies, although lasting 1 month, are typically more intensive (Traverso et al., 2015). Traverso et al. (2015), for example, asked children to take part in 12 training sessions over a period of 1 month. The well-known CogMed WM training involves 25 training sessions, from 10 up to 40 min each, administered 5 days a week for 5 weeks (Kronenberger et al., 2011; Grunewaldt et al., 2013, 2016; Hardy et al., 2016). Some of the findings of the two studies discussed in this paper (i.e., the reduced impact of the training on inhibition) might be explained by the short duration or moderate intensity of the training (see Diamond and Ling, 2016, for a discussion of the effects training duration and intensity). Future studies should test this hypothesis by comparing coding training of different duration and intensity. Interestingly, however, the short duration of our training was sufficient for children to earn significant benefits for simple and complex EFs, and to retain them after 1 month from the end of the intervention.

Our delayed post-test (follow-up) was at 4 weeks/1 month distance from the end of the training, which prevents us from drawing any conclusion about the long-term retention of the effects. Yet, a comparison with other studies that used similar follow-ups (Kronenberger et al., 2011) suggests that our training was effective. Kronenberger et al. (2011) tested the efficacy of CogMed, an intensive computerized working memory training of the duration of 5 weeks. In their study, the magnitude of children’s gains at post-training was retained only for forward digit span scores (among four verbal and spatial WM measures) at a 1-month follow-up. Given the duration and intensity of our training, maintenance of the training effects at 1 month from the end of the intervention can be regarded as a truly good outcome in terms of efficacy.

A final limitation of the present studies is the lack of information on the participants’ cognitive level or general intelligence (IQ). Although none of the participants in these studies were referred to intervention for intellectual disabilities, an assessment of the children’s IQ performance through standardized tests could have provided a better picture of the sample involved in the coding training and helped interpret the effects of the intervention. The same coding activities could have, in fact, different effects based on the initial non-verbal and/or verbal cognitive resources of a child.

## Conclusion

The studies reported in this paper show how practice with coding in school not only improves measurably children’s ability to solve (computational) problems, but it may also show transfer effects on important EFs such as planning and response inhibition. In our two studies, these effects have been observed in the period of transition to school or the first years of schooling, which has been shown to be a particularly sensitive time window for the development of EFs (Roebers et al., 2011; Macdonald et al., 2014; Magi et al., 2016; Poutanen et al., 2016). Future studies should test whether the positive effects of coding extend also to older children and whether impairing factors such as low SES may mediate the efficacy of coding interventions in school. At present, coding is increasingly becoming part of the primary school curriculum worldwide. However, little is known as yet about the effects of this new learning activity on children’s cognitive development. More research should study the learning conditions that may amplify the effects of coding on children’s EFs and thus promote children’s cognitive development. The work we are conducting aims at bridging this knowledge gap.

## Data Availability Statement

The datasets generated for this study are available on request to the corresponding author.

## Ethics Statement

The studies involving human participants were reviewed and approved by the Ethical Committee of the Department of Developmental Psychology (University of Padova, Italy). Written informed consent to participate in this study was provided by the participants’ legal guardian/next of kin.

## Author Contributions

BA contributed to study design, statistical analyses, and manuscript writing. TV contributed to study design and manuscript writing. CM and ML contributed to data collection.

## Conflict of Interest

The authors declare that the research was conducted in the absence of any commercial or financial relationships that could be construed as a potential conflict of interest.

## Footnotes

1 http://scratch.mit.edu

2 https://code.org/

3 https://programmailfuturo.it/come/lezioni-tecnologiche/corso-1
